# MuSK myasthenia gravis: from antigen–antibody to clinical translation

**DOI:** 10.3389/fimmu.2026.1732705

**Published:** 2026-07-28

**Authors:** Xinyue Zhou, Jing Zhang, Haiyan Dong, Xinxin Cui, Hanxin Zhang, Mengdi Zhang, Ruichen Liu, Qian Liu, Jia Hu, Jing Liu, Yingna Zhang, Xue Zhao, Ting Chang, Zhe Ruan, Junhong Yang, Jie Lv, Feng Gao

**Affiliations:** 1 Department of Neuroimmunology, Henan Institute of Medical and Pharmaceutical Sciences, Zhengzhou University, Zhengzhou, Henan, China; 2 Department of Neurology, Tangdu Hospital, The Fourth Military Medical University, Xi’an, China; 3 Department of Encephalopathy, First Affiliated Hospital of Henan University of Chinese Medicine, Zhengzhou, China

**Keywords:** antigen–antibody, autoimmune disease, detection and treatment, MuSK, myasthenia gravis

## Abstract

Anti-MuSK antibody–positive myasthenia gravis (MuSK-MG) is characterized by severe and potentially life-threatening weakness. Anti-MuSK antibodies are key pathogenic drivers of this disorder. This review provides a comprehensive summary of the current state-of-the-art with respect to the understanding of MuSK and its role in MuSK-MG. MuSK, a receptor tyrosine kinase, is essential at the neuromuscular junction, where it forms an agrin–LRP4–MuSK tripartite functional complex that initiates and maintains agrin-induced acetylcholine receptor clustering at the postsynaptic membrane. Although anti-MuSK antibodies encompass all IgG subclasses (IgG1–4), IgG4 is the predominant pathogenic subclass. *In vivo*, IgG4 antibodies undergo Fab-arm exchange, acquiring functional monovalency; this monovalent IgG4 disrupts LRP4–MuSK interactions, thereby blocking downstream signaling and precipitating myasthenic symptoms. Emerging evidence indicates that IgG1–3 anti-MuSK antibodies are also pathogenic, although some clones exhibit agonistic effects on MuSK phosphorylation. Further, this review examines the various diagnostic methods for this condition and weighs the advantages and disadvantages. Both MuSK protein and its autoantibodies are pivotal for diagnosis and therapy. Radioimmunoprecipitation assays and enzyme-linked immunosorbent assays and their benefits ad limitations are discussed, and cell-based assays are noted for their high accuracy. Anti-MuSK antibody testing is the gold standard diagnostic biomarker. Finally, the review covers the various therapeutic strategies for MuSK-MG, including immunosuppressants, monoclonal antibodies, CAR-T cell therapy, and neonatal Fc receptor blockade. B-cell–targeted therapies (e.g., anti-CD20 monoclonal antibodies) and MuSK agonists represent promising future therapeutic strategies for MuSK-MG. Overall, this review provides a definitive summary of the present practices and future strategies for the diagnosis and treatment of MuSK-MG, laying out clear markers for further studies that have to be undertaken to enhance our understanding of this condition.

## 1 Introduction

Myasthenia gravis (MG) is an acquired autoimmune condition driven by pathogenic autoantibodies targeting the neuromuscular junction (NMJ) endplate. This autoimmune attack compromises neuromuscular transmission, leading to the characteristic symptoms of voluntary muscle weakness and fatigue (1). Epidemiological estimates indicate a global prevalence of MG ranging from 150 to 250 cases per million, with an annual incidence of 4–10 diagnoses per million (2). In China, the reported incidence is about 0.68 per 100,000 individuals, and the disease demonstrates a female predominance. Regarding outcomes, the in-hospital mortality rate is documented at 14.69 per 1,000 admissions, primarily attributable to respiratory failure and pulmonary infections (3). Most MG cases are driven by autoantibodies against the acetylcholine receptor (AChR). In approximately 5% of patients, the disease is induced by autoantibodies targeting muscle-specific receptor tyrosine kinase (MuSK) or low-density lipoprotein receptor-related protein 4 (LRP4) (4). Patients with anti-MuSK antibodies typically present with more severe clinical manifestations and are associated with poorer prognoses (5). Anti-MuSK antibody–positive MG (MuSK-MG) predominantly affects the bulbar and ocular muscles. Approximately one-third of patients with MuSK-MG exhibit ptosis and diplopia, while over 40% manifest initial bulbar muscle involvement, including weakness of the facial, pharyngeal, and lingual muscles, often accompanied by respiratory compromise and dysphagia. Limb weakness is uncommon (6, 7). Standard therapeutic interventions for AChR-MG, such as thymectomy and cholinesterase inhibitors, are less effective in MuSK-MG. Consequently, the clinical management of MuSK-MG centers predominantly on the use of immunosuppressants, corticosteroids, or therapies that deplete B cells, including the anti-CD20 monoclonal antibody rituximab (8). The distinctive clinical presentation and therapeutic response in MuSK-MG are closely linked to the underlying pathogenic mechanisms of MuSK autoantibodies.

Compared with AChR−MG, which has been studied for decades, MuSK−MG was only discovered in 2001 after the identification of anti−MuSK antibodies in patients with seronegative MG (9). MuSK−MG has a distinct clinical phenotype, including prominent bulbar weakness, facial and neck muscle involvement, severe generalized weakness, and frequent respiratory dysfunction. In addition, it usually responds poorly to conventional symptomatic or immunosuppressive therapies, making it a clinically important subtype that warrants dedicated investigation (10).

**Figure 1:**
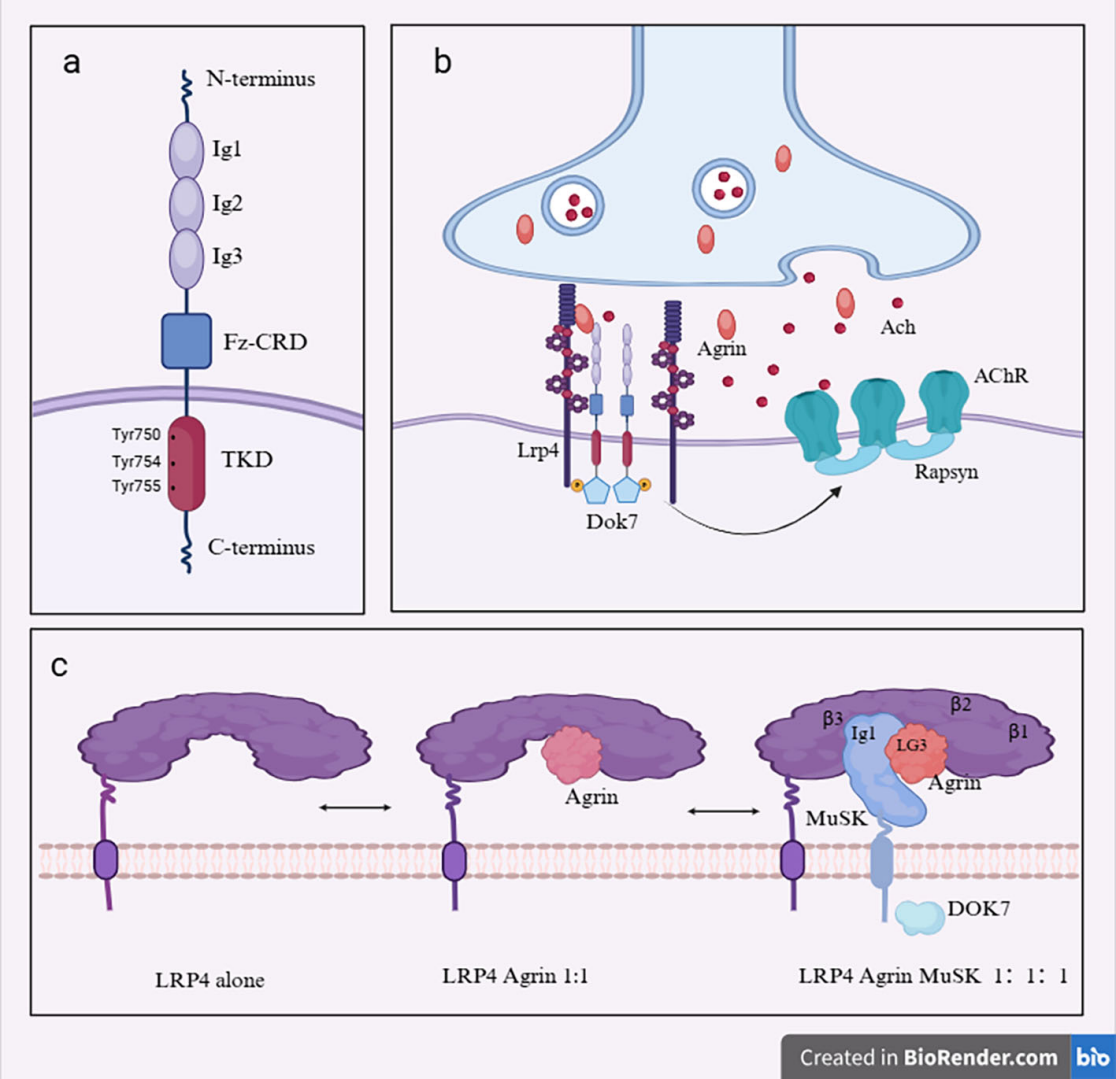
MuSK architecture and signaling at the neuromuscular junction. **(A)** MuSK is a single-pass transmembrane glycoprotein. Its extracellular domain comprises three immunoglobulin-like domains (Ig1–3), a frizzled-like domain (Fz-CRD), and a cysteine-rich domain (CRD). The intracellular tyrosine kinase domain (TKD) contains three key tyrosine residues (Tyr750, Tyr754, and Tyr755) that are phosphorylated upon activation. **(B)** Signaling cascade at the neuromuscular junction: upon co-stimulation by LRP4 and agrin, MuSK dimerizes and autophosphorylates its TKD. Activated MuSK recruits and phosphorylates Dok7, propagating downstream signaling that drives AChR clustering at the postsynaptic membrane. **(C)** Schematic diagram of MuSK and agrin LRP4 assembly on the cell surface. Edited image adapted from 10.1073/pnas.2300453120. Created with BioRender.com.

MuSK is a single-pass transmembrane protein located on the postsynaptic endplate membrane. Together with the co-localized single-pass transmembrane protein LRP4 and the synaptic vesicle–derived glycoprotein agrin released from presynaptic motor-nerve terminals, it forms the agrin–LRP4–MuSK tripartite functional complex that mediates agrin-induced clustering and maintenance of the AChR lattice at the endplate (11, 12). The pathogenesis involves anti-MuSK antibodies in positive sera binding the MuSK extracellular domain, thereby disrupting the MuSK–LRP4 interaction or the assembly of the agrin–LRP4–MuSK complex. This disruption subsequently blocks the agrin-induced phosphorylation of the MuSK intracellular domain, which is essential for the preservation of the clustered AChR scaffold at the endplate (13, 14). The agrin–LRP4 interaction is blocked *in vivo* by the binding of LRP4 antibodies to the membrane-bound receptor, thereby abolishing the subsequent clustering of AChRs at the endplate (15) (**Figure 1**). However, the pathogenic mechanism of MuSK autoantibodies remains elusive. Critical questions regarding the major immunogenic region of MuSK, its functional engagement with agrin and LRP4, and the subclass-specific effects of autoantibodies remain unanswered. In this review, we explore these key uncertainties and their translational relevance to clinical practice. Accordingly, this article systematically reviews the current state of research on MuSK-MG, from molecular mechanisms to clinical translation, with emphasis on the MuSK antigen, the pathogenic and agonistic mechanisms of anti-MuSK antibodies, detection strategies, and recent advances in clinical management.

## 2 Disease specificity of MuSK-MG: an IgG4-mediated autoimmune disease distinct from AChR-MG

Although MuSK-MG and AChR-MG are both antibody-mediated forms of myasthenia gravis, they differ substantially in immunopathogenesis, clinical phenotype, thymic pathology, and therapeutic response. Rather than being considered merely another serological subtype of MG, MuSK-MG should be recognized as a distinct IgG4-mediated autoimmune disease.

From an immunopathogenic perspective, the pathogenic antibodies in AChR-MG are predominantly of the IgG1 and IgG3 subclasses and typically impair the structure and function of the neuromuscular endplate through complement activation, receptor blockade, and antigenic modulation/internalization (16).In contrast, MuSK-MG is dominated by IgG4 antibodies. IgG4 can undergo Fab-arm exchange, generating bispecific, functionally monovalent antibody molecules (13, 17) In MuSK-MG, the principal pathogenic effect of IgG4 antibodies is not the direct destruction of the endplate membrane, but rather the disruption of the interaction between LRP4 and MuSK, thereby interfering with Agrin–LRP4–MuSK signaling and ultimately inhibiting AChR clustering and endplate maintenance (18, 19). This mode of “functional blockade” is fundamentally distinct from the “structural injury” pattern observed in AChR-MG.

This immunopathological distinction is mirrored clinically. MuSK-MG more often presents with bulbar-predominant weakness, including dysarthria, dysphagia, nasal speech, masticatory weakness, and involvement of facial, neck, and respiratory muscles, and may progress rapidly with early myasthenic crisis in some patients (6, 20, 21). It also shows a higher female predominance. In addition, unlike AChR-MG, which is frequently associated with thymic hyperplasia or thymoma, MuSK-MG rarely shows typical thymic abnormalities, consistent with the limited role of thymectomy in this subgroup (22, 23).

These differences have direct therapeutic implications. While acetylcholinesterase inhibitors often provide symptomatic benefit in AChR-MG, their efficacy is generally less satisfactory in MuSK-MG (6, 24). By contrast, MuSK-MG appears particularly responsive to B-cell-targeted therapy, especially rituximab, highlighting its antibody- and B-cell-driven disease biology (25–27). Plasma exchange also plays an important role during acute exacerbations because of its rapid antibody-clearing effect (28). Collectively, these features support the view that MuSK-MG is a distinct autoimmune disease entity with unique antibody biology, clinical characteristics, and therapeutic requirements.

Accordingly, viewing MuSK-MG simply through the traditional conceptual framework of AChR-MG risks obscuring its distinctive pathogenic mechanisms and specific clinical management requirements. A more appropriate perspective is to regard MuSK-MG as a distinct disease entity characterized by unique antibody biology, a recognizable clinical spectrum, and a differentiated therapeutic strategy. Establishing such a disease-specific framework is important not only for explaining why MuSK antibodies produce pathogenic effects different from those of AChR antibodies, but also for advancing the clinical transition from empirical treatment to mechanism-based precision medicine.

To highlight the systematic differences between MuSK-MG and AChR-MG in immunopathology, clinical features, and treatment response, we first present a comparative overview of their key distinctions (**Table 1**). As shown in **Table 1**, the differences between the two conditions are not limited to the target antigen itself, but extend across antibody subclass composition, pathogenic mechanisms, clinical phenotype, thymic pathology, and therapeutic responsiveness. These observations further support the need to discuss MuSK-MG separately as an autoimmune disease with a distinct biological identity.

**Table 1:** Comparison of clinical and immunological characteristics between MuSK−MG and AChR−MG patients.

Characteristic	MuSK-MG	AChR-MG
Proportion of MG patients	~5–10%	~80–85%
Predominant IgG subclass	Mainly IgG4, with possible IgG1-3	Mainly IgG1/IgG3
Key immunological features	IgG4 can undergo Fab-arm exchange, forming functionally monovalent antibodies; mostly complement-independent	Complement activation is common; antibodies can cross-link receptors and promote internalization
Main pathogenic mechanisms	Interference with LRP4-MuSK interaction, disruption of the Agrin-LRP4-MuSK signaling axis, leading to impaired AChR clustering	Complement-mediated damage to the endplate, AChR cross-linking and internalization, and receptor functional blockade
Clinical phenotype	Bulbar muscle involvement is more prominent	Both ocular and generalized forms are common; proximal limb weakness is frequent
Thymic pathology	Thymic hyperplasia/thymoma are rare	Thymic hyperplasia is relatively common, some with thymoma
Value of thymectomy	Usually limited; not a routine strategy	Clear benefit in some patients
Response to cholinesterase inhibitors	Limited efficacy or intolerance	Beneficial for most patients
Response to plasma exchange	Often good, especially in acute exacerbations	Also effective
Response to rituximab	Usually good; may be considered earlier	Effective, but overall heterogeneity is greater

## 3 MuSK and anti-MuSK antibodies

### 3.1 MuSK: from structure to function

The receptor tyrosine kinase MuSK, essential for NMJ integrity, has a molecular weight of 100 kDa and consists of 869 amino acids. Originally discovered by Jennings et al. via PCR-based screening of tyrosine kinases in *Torpedo californica*, the *MUSK* gene is located on chromosome 9. Interestingly, MuSK is not restricted to skeletal muscle but is also expressed in excitatory neurons of the mammalian central nervous system (29). Structurally, MuSK is a type I transmembrane glycoprotein with a single membrane-spanning region. Its extracellular portion comprises three immunoglobulin-like domains (Ig1–3) and a cysteine-rich domain (CRD), complemented by an intracellular tyrosine kinase domain (TKD) in its cytoplasmic tail (30). MuSK’s extracellular region displays a modular architecture, beginning with two highly homologous immunoglobulin domains (Ig1 and Ig2) in tandem. This configuration is extended by an Ig2–Ig3 linker that mirrors the structure of the preceding Ig1–Ig2 linker 16. Stiegler et al. showed that the first two Ig-like domains (Ig1–2) of MuSK are primarily responsible for agrin-induced AChR clustering, and this interaction was resolved at a resolution of 2.2 Å using cryo-electron microscopy (cryo-EM) (31). The Ig1 domain of MuSK interacts with both agrin and LRP4. Subsequent activation of its tyrosine kinase activity drives the formation and maintenance of the AChR-rich postsynaptic apparatus and ensures correct positioning of the developing nerve terminal (32–35). Although the Ig1 domain of MuSK is critical for LRP4 binding (34), prior structural knowledge of the agrin–LRP4–MuSK signaling complex was limited, hindering a mechanistic understanding of why MuSK requires both ligands for activation. To elucidate this, Xie et al. (2023) determined the cryo-EM structure of the ternary complex (1:1:1) at 3.8 Å resolution. The unveiled structure shows LRP4’s N-terminal β-propeller domains forming an arc-shaped scaffold, with agrin and MuSK nestled within its concave surface. In this configuration, the LRP4 arc acts as a clamp that pre-organizes and secures the ligands, markedly boosting their binding affinity. These findings provide a structural basis for the complex’s assembly and the prerequisite of dual ligand engagement for MuSK activation (12).Although the structure of the Agrin–LRP4–MuSK ternary complex has been resolved, several key questions remain unanswered, including the spatiotemporal propagation of downstream signaling following MuSK activation, the dynamic conformational changes induced by Dok-7 binding to MuSK, and the negative regulators of AChR clustering. These knowledge gaps limit a comprehensive understanding of the pathogenesis of MuSK-MG.

Studies employing radioimmunoprecipitation (RIPA) and enzyme-linked immunosorbent assay (ELISA) have demonstrated a robust correlation between MuSK antibody titers and clinical severity, with these antibodies mapping to the MuSK-Ig1 domain (36–38). This indicates that at least a subset of anti-MuSK antibodies in patients exerts pathogenicity by binding to the Ig1 region, thereby disrupting downstream signaling. Although the immunodominant epitope(s) of MuSK have not yet been comprehensively identified, these data strongly suggest that the Ig1 domain constitutes the principal immunogenic region; however, the exact residues involved remain to be identified.

In 2025, Fish et al. at Brown University employed a MuSK-Ig3-deficient mouse model to demonstrate that the Ig3 domain is indispensable for maintaining postsynaptic Nav1.4 density, thereby amplifying cholinergic signaling and ensuring the muscle excitability required for action potential firing (39). However, the precise function and mechanistic roles of the Ig2 domain remain unclear.

The frizzled-like (Fz)-CRD contains 10 conserved cysteine motifs stabilized by a distinctive disulfide bond pattern (40). This domain is indispensable for agrin-induced MuSK activation and AChR clustering. The crystal structure of the MuSK Fz-CRD, solved at a resolution of 2.1 Å, reveals striking homology to the CRDs of the Wnt receptor Frizzled-8 (Fz8) and the secreted Frizzled-related protein-3 (sFRP3), suggesting that the Fz-CRD may facilitate cell-surface MuSK dimerization (41–43). Halliez et al. (2025) demonstrated that antibodies targeting the MuSK-CRD domain disrupt the agrin–LRP4–MuSK signaling axis by specifically weakening the MuSK–LRP4 interaction, thereby inhibiting AChR clustering. This mechanistic insight was gleaned from a study employing both passive and active EAMG (experimental autoimmune myasthenia gravis) models. The functional consequence of this signaling blockade was the induction of classic myasthenic symptoms, including muscle weakness with neurotransmission fatigue and profound structural defects at the NMJ (44).

The intracellular TKD comprises a 52–amino acid juxtamembrane region, a canonical kinase domain containing three tyrosine residues (Tyr750, Tyr754, and Tyr755) within the activation loop, and an eight–amino acid C-terminal tail. Four tyrosines—three in the activation loop and one in the juxtamembrane region—constitute the principal phosphorylation sites of MuSKs (18). Upon agrin release from the motor nerve terminal, agrin-dependent MuSK activation drives receptor dimerization and trans-autophosphorylation of Tyr553 within the juxtamembrane segment. This event disrupts the inhibitory juxtamembrane conformation that normally prevents activation-loop phosphorylation, allowing subsequent trans-phosphorylation of the activation-loop tyrosines (Tyr750, Tyr754, and Tyr755) and achieving full kinase activation. However, the precise mechanism by which the kinase domain itself dimerizes to trigger activation remains incompletely defined (45).

In addition to their role in MuSK-MG, pathogenic variants of the *MUSK* gene give rise to a distinct form of neuromuscular transmission failure known as congenital myasthenic syndrome (CMS) (46). Maselli et al. showed that missense mutations in *MUSK* can cause severe CMS; mutant MuSK proteins fail to interact with Dok-7, while retaining their ability to bind to LRP4 and Tid1. The inability to recruit Dok-7 is the principal molecular defect underlying CMS that is attributable to MuSK mutations (47).

**Figure 2:**
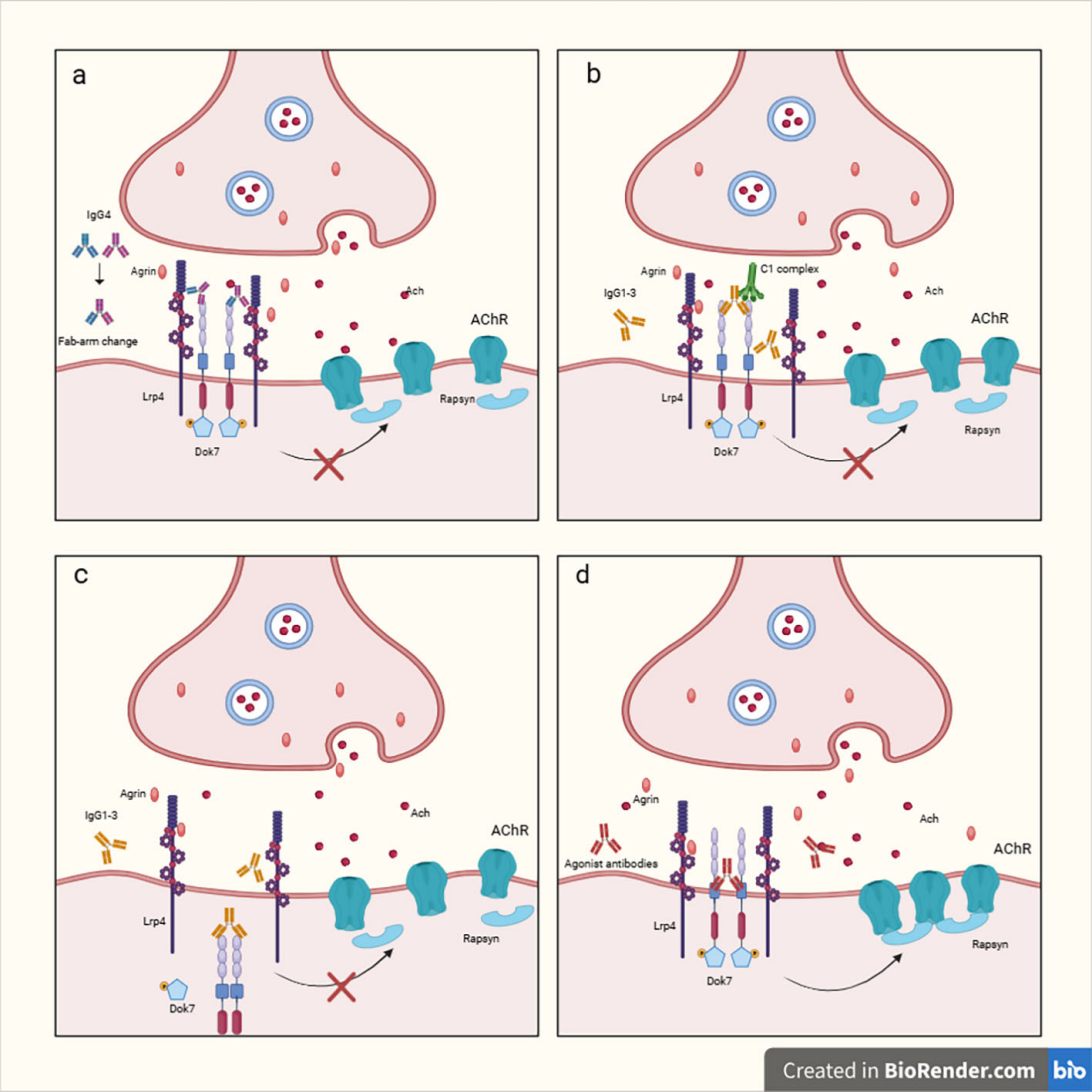
Mechanisms of anti-MuSK antibody action. **(A)** In MuSK-MG, IgG4 antibodies undergo Fab-arm exchange to become bispecific and bind MuSK monovalently; the resulting Fab fragments and intact IgG4 disrupt the LRP4–MuSK interaction, thereby preventing AChR clustering. **(B)** IgG1–3 subclass antibodies may activate the complement cascade, leading to tissue damage. **(C)** Bivalent engagement by these antibodies can trigger MuSK internalization and subsequent downregulation of membrane expression. **(D)** Agonist antibodies bind MuSK, enforce receptor dimerization, and stimulate MuSK phosphorylation independent of its natural ligands. Created with BioRender.com.

MuSK serves as a central hub of endplate signal transduction. It is indispensable not only for the establishment of the neuromuscular junction (NMJ) during embryonic development, but also for the maintenance of endplate architecture in adulthood. Animal studies have shown that MuSK deficiency leads to failure of AChR clustering and defective NMJ formation, underscoring its essential role in neuromuscular transmission (19). This also helps explain why autoantibodies targeting MuSK, even in the absence of prominent complement-mediated injury, can still produce severe weakness through a mechanism of “signal blockade”. **Figure 2** represents MuSK architecture and signaling at the NMJ.

From a structure–function perspective, antibody binding to different MuSK domains may lead to distinct functional consequences. Previous studies suggest that the Ig-like 1 domain is closely involved in the LRP4–MuSK interaction; accordingly, antibodies targeting this region are more likely to exhibit classical antagonistic effects. Antibodies recognizing other MuSK domains, including the Fz-CRD, may have distinct functional effects ^(^31, 48, 49^).^ Therefore, a deeper understanding of the domain-specific functions of MuSK will not only help explain the pathogenic heterogeneity of patient-derived antibodies, but also provide a theoretical basis for the development of mechanism-corrective therapeutic strategies.

### 3.2 Anti-MuSK antibodies: from antigen to antibody

MuSK-MG is caused by B-cell–derived autoantibodies directed against MuSK that disrupt the NMJ. Although the dominant subclass is IgG4 (50), Cao et al. at the University of Oxford established that IgG1–3 antibodies are also similarly pathogenic (51).

#### 3.2.1 Primary pathogenic mechanisms mediated by IgG4 antibodies

MuSK-MG falls within a distinct category of IgG4-mediated autoimmune diseases (IgG4-AID), characterized by pathogenic autoantibodies of the IgG4 subclass. This family includes conditions like pemphigus vulgaris, thrombotic thrombocytopenic purpura, and several neurological and renal autoimmune disorders. While each disease targets a unique autoantigen and produces a different syndrome, they are all strikingly similar in their genetic background, disease-driving mechanisms, clinical evolution, and treatment responses (52). Kolfschoten et al. demonstrated that IgG4 antibodies are dynamic molecules—they undergo Fab-arm exchange, in which a heavy/light-chain half-molecule swaps with a corresponding half-molecule from another IgG4 to generate bispecific, functionally monovalent antibodies (53). In MuSK-MG, patient-derived IgG4 antibodies engage in this exchange, binding MuSK in a monovalent fashion (54). Koneczny et al. confirmed the pathogenicity of Fab-arm–exchanged antibodies. They reported that Fab fragments and IgG4 MuSK antibodies block the LRP4–MuSK interaction and reduce agrin-induced AChR clustering in C2C12 myotubes (54). The disruption of LRP4–MuSK binding by IgG4 antibodies is an established pathogenic mechanism in MuSK-MG (55).IgG4 antibodies are functionally monovalent and have limited capacity to activate the complement cascade; accordingly, they are generally unable to induce complement C3 activation or membrane attack complex (MAC) formation (56).

However, Oskam et al. showed that IgG4 can bind C1q, albeit with lower avidity than IgG1, and activate complement via the classical pathway under high antigen and antibody concentrations. Importantly, bispecific monovalent IgG4 generated by Fab-arm exchange exhibits a weaker complement activation ability than monospecific IgG4, implying that IgG4-mediated complement activation could contribute to pathology, although it is unlikely to play a role in MuSK-MG (57).

#### 3.2.2 Non-canonical pathogenic mechanisms of IgG1–3

Koneczny et al. demonstrated that IgG1–3 subclasses are bivalent and could, theoretically, trigger MuSK endocytosis. However, at an antibody titer of 0.17 nM, no internalization was detected in HEK293 cells transfected with a MuSK plasmid, suggesting that either the epitope recognized by IgG1–3 antibodies does not permit inter-MuSK cross-linking or additional factors are required for endocytosis (55). Conversely, Cole et al. employed IgG from a patient with a high MuSK titer in both cellular and animal paradigms and showed that patient antibodies induced MuSK activation followed by rapid internalization, leading to postsynaptic MuSK depletion and disassembly of the AChR scaffold, which was unequivocally confirmed in C2C12 myotube cultures (58). The apparent discrepancy between these studies likely arises from several variables. First, the cell type used in these studies. It is important to note that C2 myotubes natively express MuSK-interacting partners (e.g., LRP4, Dok-7), potentially furnishing a micro-environment conducive to endocytosis. Second, the antibody characteristics also play a role. The patient-derived antibodies used by Cole et al. may target distinct epitopes (e.g., within the CRD) that are more efficient at triggering receptor internalization. Finally, concentration effects should also be taken into account. Cole et al. used supra-physiological antibody concentrations, which could amplify otherwise marginal endocytic events. Although endocytosis is not considered the dominant pathogenic mechanism in MuSK-MG, certain antibody subclasses or niche microenvironments may represent exceptions (51).These possibilities warrant systematic validation.

IgG1, IgG2, and IgG3 subclasses activate the complement cascade, thereby mediating tissue injury and also reducing membrane expression of their target antigen by driving internalization through bivalent engagement (59). In 2013, Koneczny et al. established that although MuSK-specific IgG1–3 antibodies do not block LRP4–MuSK binding, they potently inhibit agrin-induced AChR clustering. These antibodies decrease AChR density and precipitate neuromuscular dysfunction in patients with MuSK-MG (55). Expanding on this premise, researchers at Leiden University produced recombinant MuSK antibodies in 2019, sourced from clonal B cells of patients. The isolated monoclonal sequences, belonging to the IgG1 and IgG3 subclasses, were found to be specifically directed against the MuSK Ig-like 1 domain and capable of binding the NMJ. Strikingly, these bivalent monospecific MuSK antibodies promoted MuSK autophosphorylation and triggered AChR clustering to a certain extent, independent of agrin. Together, these results suggested that MuSK antibodies derived from patients can exhibit both agonistic and antagonistic functional properties, likely determined by the specific epitope they engage (14). In the same year, Takata et al. employed fluorescently labeled MuSK antigen tetramers to isolate MuSK-reactive B cells from six patients with MuSK-MG and subsequently generated a panel of human monoclonal autoantibodies (mAbs). Three highly specific mAbs—two of the IgG4 subclass and one of IgG3—were selected for detailed characterization; all three recognized epitopes were within the MuSK Ig-like domains. Although these mAbs potently inhibited AChR clustering, they paradoxically enhanced MuSK phosphorylation, unveiling an alternative, yet undefined, mechanism of AChR cluster suppression (60). In 2023, Cao et al. at the University of Oxford purified IgG1–3 fractions from the plasma of a patient with MuSK-MG and demonstrated that MuSK-IgG1–3 antibodies impair AChR clustering without blocking agrin-induced MuSK phosphorylation. Moreover, the canonical downstream pathway—MuSK-mediated phosphorylation of βAChR via DOK7—remained intact. However, IgG1–3 exposure abolished mature AChR clusters and reduced both AChR microclusters and surface-expressed receptors. Strikingly, the SHP2 inhibitor NSC-87877 restored microcluster formation and promoted the assembly of full AChR clusters (51).In 2026, Masi Gianvito et al. drew an analogy between the mechanism of action of IgG1–3 antibodies and the newly discovered bivalent IgA antibodies. Monoclonal IgA (similar to IgG1–3) acts as an agonist, inducing phosphorylation and clustering, but when multiple bivalent clones are combined (mimicking serum polyclonality), they lead to severe clustering defects. This suggests that the pathogenicity of IgG1–3 may likewise depend on inter−clonal synergy rather than merely on the level of phosphorylation (61). Collectively, these findings underscore that the precise pathogenic mechanism of MuSK-IgG1–3 monoclonal antibodies is not yet fully understood and warrants further investigation.

### 3.3 MuSK antibody activation

Unlike the AChR antibodies in MG, which are exclusively pathogenic, a subset of anti-MuSK antibodies functions as agonists. These agonist antibodies bind to the Fz -CRD domain of MuSK, enforce receptor dimerization, and stimulate MuSK phosphorylation independent of LRP4, bypassing the canonical agrin-dependent pathway (62). Such agonist-driven signaling is emerging as a promising therapeutic modality for a range of neuromuscular and neurodegenerative conditions (63).Currently reported MuSK agonistic antibodies primarily target the Fz-CRD domain (e.g., ARGX-119). However, some studies have shown that certain monoclonal antibodies directed against the Ig1 domain can also exert partial agonistic effects, including enhanced MuSK phosphorylation. These findings suggest that agonistic activity may not be restricted to a single domain (14, 64).

In 2023, investigators at the Leiden University Medical Center generated 15 humanized variants and five camelid-derived MuSK nanobodies from patient-derived parent clones, all engineered to target the MuSK Ig1 domain as agonistic monoclonal antibodies. These constructs effectively activated MuSK and promoted AChR clustering in cultured myotubes. Nevertheless, when administered to NOD/SCID mice subjected to passive IgG4 MuSK-MG induction, MuSK agonists induced accelerated weight loss and failed to ameliorate myasthenic symptoms (65). Despite this discouraging preclinical observation, a growing body of evidence indicates that agonistic anti-MuSK antibodies can alleviate the clinical manifestations of MuSK-MG, highlighting the need for further mechanistic refinement and context-optimized therapeutic strategies.

In 2024, Vanhauwaert et al. generated and characterized ARGX-119, a humanized agonist monoclonal antibody directed against the Fz-CRD domain of MuSK that was developed for therapeutic applications in neuromuscular disorders. ARGX-119 has a high binding affinity to the Fz-CRD domain of MuSK from humans, non-human primates, rats, and mice, and exhibits no off-target binding, rendering it suitable for further clinical development. Mechanistically, ARGX-119 activates MuSK without interfering with neural agrin, which is its natural ligand, and promotes dose-dependent AChR clustering, thereby stabilizing neuromuscular function. ARGX-119 is a promising therapeutic option for alleviating neuromuscular diseases characterized by compromised synaptic transmission. However, further clinical development is warranted (49). Subsequently, the same team investigated whether ARGX-119 could ameliorate the disease in a passive immunization model induced by patient-derived polyclonal IgG4. The results demonstrated that ARGX-119 improved survival and attenuated muscle weakness in mice challenged with patient material. This first-in-class proof-of-concept study in a clinically relevant MuSK-MG model established a foundation for therapeutic development of ARGX-119 for neuromuscular diseases (66). In parallel, Oury et al. at Stanford Medicine passively immunized mice with autoantibodies from patients with MuSK-MG, which resulted in the induction of severe neuromuscular deficits. Remarkably, these deficits were reversed by the administration of the MuSK agonist antibody ARGX-119, offering a selective and direct disease-targeting alternative to systemic immunosuppression for the treatment of MuSK-MG (67).

Agonist antibodies that promote MuSK activation can safeguard NMJ integrity, and ARGX-119 exemplifies this therapeutic potential of agonist antibodies for disorders characterized by synaptic dysfunction. Notably, the reported findings on MuSK agonistic antibodies remain inconsistent. These discrepancies may be attributable to differences in antibody origin, epitope specificity, valency, as well as the experimental systems used and the stage of disease examined. Nevertheless, all future agonist-antibody programs must be engineered to guarantee a safe therapeutic window, rigorously excluding off-target activation, inadvertent complement engagement, and receptor overstimulation, which could cause adverse neuromuscular events. Therefore, future studies should move beyond the simple question of whether anti-MuSK antibodies are pathogenic and instead aim to establish a more refined correspondence among antibody subclass, targeted domain, functional effect, and clinical phenotype. The mechanisms through which anti-MuSK antibodies exert their effects are summarized in **Figure 2**.

## 4 Clinical applications

### 4.1 Anti-MuSK antibody testing—diagnosis of MuSK-associated MG

MuSK-MG is diagnosed based on the presence of fluctuating muscle weakness, which is typical of MG, and when any one of the following three criteria is met (1): pharmacological testing (2), characteristic electrophysiological findings, or (3) detection of anti-MuSK antibodies in serum (68). Serological testing for anti-MuSK antibodies is a pivotal diagnostic biomarker. Available assays include RIPA, ELISA, and cell-based assays (CBA).

In RIPA, radiolabeled recombinant MuSK is incubated with patient serum and immune complexes are precipitated and quantified by scintillation counting. Owing to its high sensitivity and specificity, RIPA remains the gold standard for the detection of anti-MuSK antibody (50). For ELISA, MuSK antigen is coated onto microtiter plates. After serum incubation and enzyme-conjugated secondary antibody detection, the absorbance values are used to calculate quantitative antibody titers. ELISA is rapid, safe, and amenable to high-throughput screening; however, its sensitivity is slightly inferior to that of RIPA, and low-titer samples may yield false-negative results In CBAs, HEK293 cells ectopically expressing MuSK are fixed onto slides, exposed to patient serum, and visualized using fluorochrome-conjugated secondary antibodies. Fluorescence microscopy and flow cytometry allow the direct visual confirmation of antibody binding, thus conferring high specificity.

In 2019, our laboratory established an in-house MuSK-CBA protocol and benchmarked its performance against RIPA, ELISA, and commercial CBAs. Among the 251 AChR-Ab-negative sera, 46 (18.3%) were positive for MuSK-CBA, whereas only 4 of 624 (0.6%) AChR-Ab-positive samples showed positive results. Our high-specificity CBA demonstrated a sensitivity superior to that of commercial ELISA and fixed-cell CBA (MuSK-direct immunofluorescence), underscoring its diagnostic utility (69).

In 2025, Mengfei et al. demonstrated that anti-MuSK fixed CBAs exhibited excellent positive predictive values (PPV) for MG, supporting their use as a first-line test in clinical practice for suspected MG (70). In 2023, Kwon et al. showed that, among 89 patients with AChR antibody–negative generalized MG, ELISA detected MuSK antibodies in 22 cases (24.7%), CBA in 25 cases (28.1%), and RIPA in 14 of 51 samples (27.5%), confirming the diagnostic accuracy and clinical utility of MuSK-ELISA in patients with MG (71). In 2024, the Institut de Recerca Biomèdica Sant Pau (Sant Pau Biomedical Research Institute) at the Universitat Autònoma de Barcelona distributed 13 patient samples to 16 laboratories for inter-laboratory comparison. The analysis revealed that ELISA displayed a sensitivity of 31.3% and specificity of 100%, whereas CBA achieved a sensitivity of 37.5% and specificity of 100% (72). Although CBA and RIPA perform excellently in detecting anti-MuSK antibodies, their reliance on radioisotopes or genetically engineered cells may limit their accessibility in many regions. Consequently, commercially available anti-MuSK ELISA kits serve as practical alternatives (71). In 2022, Spagni et al. employed live cell–based assays (L-CBA) to screen a subset of patients with radioimmunoassay (RIA) double-seronegative MG (dSN-MG) for AChR and MuSK antibodies. Their results demonstrated that L-CBA offers a distinct advantage in the serological diagnosis of MG, particularly RIA-dSN, by enabling an approximately 8% increase in antibody detection. Moreover, L-CBA exhibits superior sensitivity to MuSK antibodies, making it a valuable tool for identifying difficult cases that return negative results on RIA and fixed CBA (73, 74).The use of L-CBA has facilitated the identification of low-affinity or conformation-dependent autoantibodies in a subset of patients with dSN-MG. Further optimization of assay sensitivity and antigen presentation may help reduce the proportion of dSN-MG cases and provide a basis for more precise treatment in these patients. A comparison of the currently available methods for anti-MuSK antibody detection is presented in **Table 2**.

**Table 2:** Comparison of laboratory methods for the detection of MuSK antibodies in myasthenia gravis.

Method	Principle	Advantages	Limitations	Clinical value in MuSK-MG
RIPA	Immunoprecipitation of patient serum antibodies with radiolabeled antigen	Reference method; high specificity	Requires radioactive facility; limited preservation of conformational epitopes	Standard for routine detection and confirmation
ELISA	Binding of patient serum antibodies to solid-phase antigen	Simple operation; suitable for high-throughput screening	Antigen conformation may be altered; limited sensitivity	Useful for initial screening or as a supplementary test
Fixed-cell CBA	Detection using MuSK expressed on the surface of fixed cells	Relatively good preservation of conformation; high specificity	Fixation may affect epitope exposure	Supplementary value for challenging cases
Live-cell CBA	Detection using MuSK expressed on the surface of living cells	Better preservation of native conformation; sensitive to low-titer antibodies	Technically demanding; lack of standardization	High value in cases with strong clinical suspicion but negative by conventional methods
Flow cytometry-based assay	Quantitative detection of antibody binding on a flow cytometry platform	Facilitates quantification and standardization	Still limited in routine application	Potential for future stratification and monitoring

Previous studies have reported that MuSK antibody titers measured using RIA or ELISA correlate well with clinical severity and outcome and that these antibodies map to the MuSK-Ig1 region (36). In 2025, Jan-Hendrik Stahl (USA) conducted a 10-year retrospective analysis of 749 serum samples from 641 patients, of whom 21 were MuSK-positive. Among the 7 patients with MuSK-MG (3% of the cohort), no significant correlation was observed between MuSK antibody titers and clinical severity (Besinger score), challenging the long-held view that MuSK antibody levels predict disease intensity (75). These findings underscore the need for further investigations incorporating mechanistic insights into antibody function to clarify this discrepancy.

Detection of anti-MuSK antibodies is an important basis for the diagnosis of MuSK-MG. Differences among the available testing methods are not merely a matter of “higher or lower sensitivity,” but also, to some extent, reflect the intrinsic heterogeneity of MuSK antibodies. Because MuSK-related autoantibodies often recognize native conformational epitopes, assay platforms that better preserve the natural conformation of membrane proteins, such as cell-based assays (CBAs), particularly live CBAs, are of greater value in diagnostically challenging cases (74, 76, 77). In the future, further classification of anti-MuSK antibodies according to IgG subclass, target domain, and functional properties may help establish a more clinically meaningful stratified diagnostic system, thereby providing a basis for prognostic assessment and individualized treatment.

### 4.2 Treatment for MuSK-MG: from conventional immunosuppressants to precision targeted therapy

MuSK-MG is a rare but typically more severe MG subtype. The therapeutic strategy for MuSK-MG should be based on its distinct immunopathogenic mechanisms rather than simply extrapolated from the treatment paradigm used for AChR-MG. Accordingly, the response patterns of MuSK-MG to acetylcholinesterase inhibitors, thymectomy, plasma exchange, IVIG, and B cell–targeted therapies differ substantially from those observed in AChR-MG. Most patients exhibit limited or no response to anticholinesterase drugs, and these agents frequently provoke adverse effects (6, 78). Corticosteroids remain the cornerstone of therapy, usually providing rapid and effective symptom control. However, their prolonged use is accompanied by substantial side effects. Traditional immunosuppressants, including azathioprine, mycophenolate, tacrolimus, methotrexate, and cyclosporine, are often employed as corticosteroid-sparing agents; however, monotherapy rarely achieves complete control. Approximately 10–15% of patients with MuSK-MG fail to respond to conventional regimens or relapse during tapering. In those with life-threatening symptoms or rapid deterioration, high-dose prednisone combined with plasmapheresis should be instituted; intravenous immunoglobulin may also be considered (78, 79).

MuSK-MG is a B-cell–mediated disorder driven by well-characterized autoantibodies of defined IgG subclasses with multiple distinct modes of action, making it an ideal target for precision therapies (80). Compared with traditional immunosuppressive regimens, B-cell–directed strategies promise more rapid and specific clinical improvement while minimizing adverse effects and enhancing patient adherence. Over the past decade, such precision approaches have entered the MuSK-MG therapeutic landscape (81). Rituximab, a chimeric anti-CD20 monoclonal antibody widely used in malignant and autoimmune diseases, binds to CD20 and triggers B-cell depletion via direct pro-apoptotic signaling, complement activation, and antibody-dependent cellular cytotoxicity, thereby reducing anti-MuSK antibody titers (82–84). Accumulating evidence has demonstrated that the early administration of rituximab in MuSK-MG yields superior outcomes, markedly improving symptoms, and reducing dependence on glucocorticoids. A clinical series has consistently reported higher response rates to rituximab in MuSK-MG, thereby increasing the likelihood of achieving sustained remission (25, 26, 85). Beyond rituximab, a spectrum of next-generation B-cell–targeted monoclonal antibodies is under active investigation, including CD20-directed agents (e.g., ofatumumab), CD38-targeting antibodies (e.g., mezagitamab), and CD40 pathway blockers (e.g., iscalimab). Anti-CD19 monoclonal antibodies (e.g., inebilizumab) act earlier during B-cell maturation and may synergize with anti-CD20 therapies (79, 86). In addition to direct B-cell depletion, BAFF-targeted strategies have been explored. Belimumab, a BAFF receptor antagonist, sequesters soluble BAFF, disrupts BAFF–BAFF-R signaling, and promotes B-cell apoptosis (87). However, in a phase 2, placebo-controlled multicenter double-blind trial (NCT01480596), the addition of belimumab to standard-of-care therapy did not meet the efficacy endpoints in patients with generalized MG (88).

Advanced therapeutic strategies now focus on monoclonal antibodies that target molecules involved in B-cell activation and B cells at distinct maturational stages, especially plasmablasts, and, more precisely, B cells that exclusively secrete anti-MuSK antibodies (89). Among these, chimeric antigen receptor (CAR) T-cell therapy offers the prospect of unprecedented specificity and efficacy. B-cell–depletion strategies employing anti-CD19 and anti-BCMA CAR-T cells are expected to provide durable remission (59). In a recent study (NCT04561557), two highly refractory, frequently relapsing patients with MG—one with AChR-MG and one with MuSK-MG—received BCMA-directed CAR-T cells. This therapy exhibited excellent safety and sustained clinical improvement over 18 months (90, 91). Chimeric autoantibody receptor T cells (CAAR-T cells) constitute a more sophisticated cellular immunotherapy strategy. This approach involves engineering T cells with a chimeric receptor that fuses the MuSK ectodomain to intracellular CD137–CD3ζ signaling domains, enabling them to precisely target and lyse B cells expressing anti-MuSK B-cell receptors. Pre-clinical evidence confirms that MuSK-CAAR-T cells selectively deplete the pathogenic anti-MuSK antibody repertoire while sparing normal B cells, and they exhibit robust therapeutic efficacy even without prior lymphodepleting conditioning (92).

**Figure 3:**
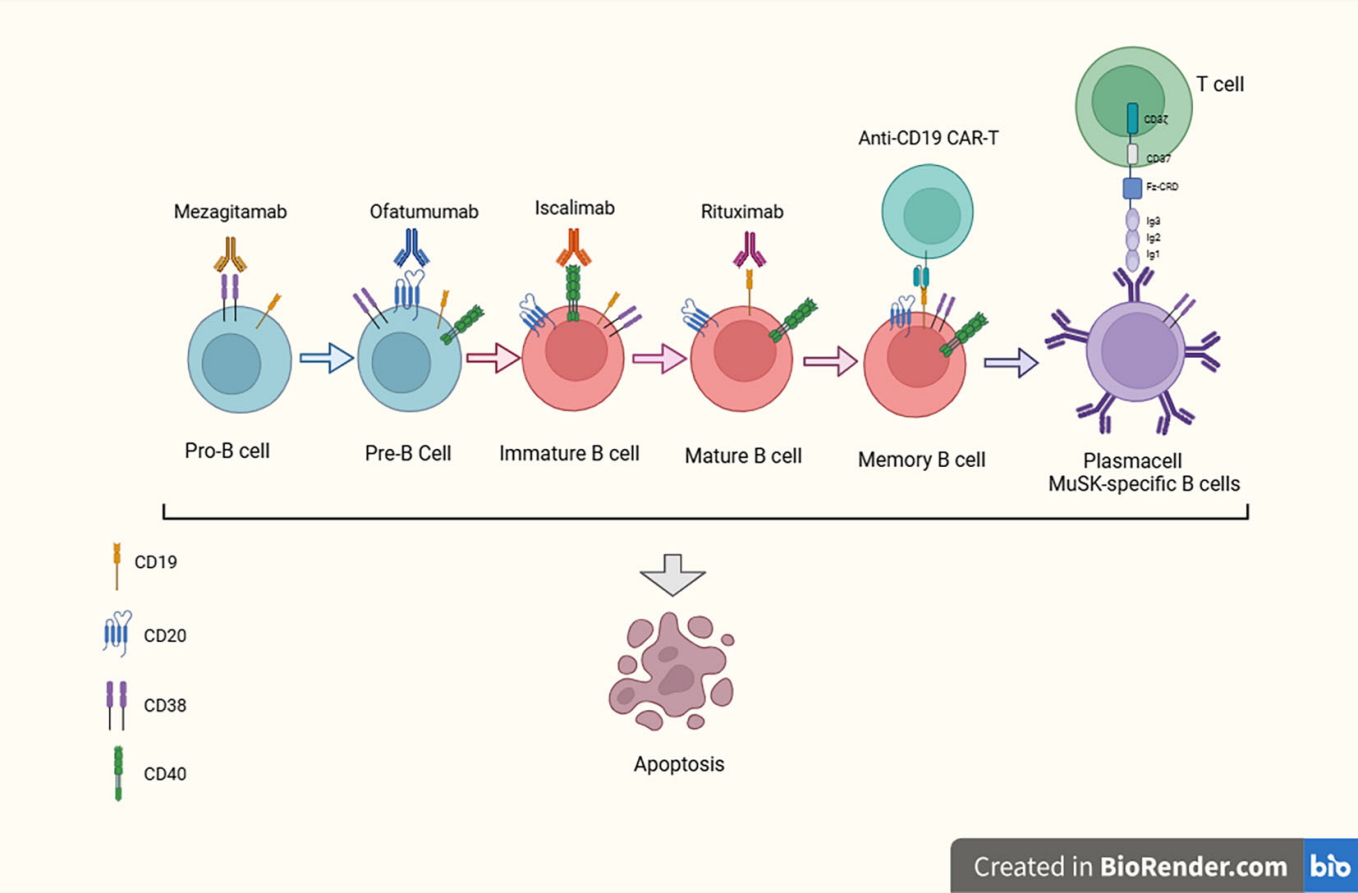
Development of MuSK antibody–secreting B cells and targeted therapy. Created with BioRender.com.

The neonatal Fc receptor (FcRn) protects IgG from lysosomal degradation. Hence, FcRn blockade accelerates IgG catabolism and rapidly lowers circulating IgG and pathogenic autoantibodies. This strategy has been proven effective in AChR-MG and is poised as a novel therapeutic avenue for MuSK-MG. Orilanomab (SYNT001/ALX1830) and rozanolixizumab (UCB7665) are humanized, high-affinity anti-FcRn IgG4P monoclonal antibodies engineered to reduce pathogenic IgG in autoimmune and alloimmune disorders (93, 94). Recently, the FcRn inhibitors, efgartigimod and rozanolixizumab, have entered the therapeutic landscape for generalized MG. A 2025 update confirmed that the FcRn blockade is both effective and safe in generalized MG, although its efficacy may depend on disease activity at the time of treatment. The successful transition from intravenous to more convenient subcutaneous formulations indicates that future demand for subcutaneous FcRn inhibitors is likely to rise (95, 96). To better illustrate the evolving therapeutic landscape of MuSK-MG, from conventional immunosuppressive approaches to precision targeted interventions, the major treatment strategies, their mechanisms of action, and clinical positioning are summarized in **Table 3**.Various B cell therapeutic strategies are shown in **Figure 3**.

**Table 3:** Comparison of therapeutic strategies for MuSK-positive myasthenia gravis.

Therapeutic strategy	Main mechanism	Features in MuSK-MG	Main advantages	Main limitations/considerations
Cholinesterase inhibitors	Increase acetylcholine concentration in the synaptic cleft	Often limited efficacy; some patients intolerant	Rapid onset; short-term symptomatic relief	Cannot correct core immune mechanisms
Glucocorticoids	Broad-spectrum immunosuppression	Commonly used as baseline therapy	Relatively rapid response	Significant long-term adverse effects
Conventional immunosuppressants	Inhibit lymphocyte activation and proliferation	Used for maintenance and steroid-sparing	Facilitate long-term disease control	Slow onset; high inter-individual variability
Plasma exchange (PE)	Remove circulating pathogenic antibodies	Often effective in acute exacerbations and bulbar/respiratory involvement	Rapid onset of effect	Invasive; resource-intensive
Intravenous immunoglobulin (IVIG)	Multi-pathway immunomodulation	Can be used for acute exacerbations or as bridging therapy	Relatively convenient	Less stable efficacy than PE in some cases
Rituximab	Deplete CD20^+^ B cells	Generally shows good efficacy in MuSK-MG	High mechanistic match; may reduce relapses	Retreatment strategy still needs optimization
CD19/CD38/BAFF-targeted therapies	Broader intervention on B-cell/plasma cell lineages	Potential direction for refractory MuSK-MG	Theoretically more comprehensive effects	Mostly in research or early-phase clinical stages
FcRn inhibitors	Accelerate IgG degradation	Theoretically attractive for antibody-mediated MuSK-MG	Rapid reduction of IgG levels	Limited specific evidence in MuSK-MG
MuSK agonist antibodies	Directly activate MuSK signaling	Novel mechanism-correcting approach	Potential to restore endplate function	Preclinical results not fully consistent
CAAR-T/CAR-T	Targeted depletion of pathogenic B cells/plasma cells	Potential highly specific option for refractory MuSK-MG	Possible etiological intervention	Still in exploratory stage

As our mechanistic understanding of MuSK-MG deepens, the therapeutic landscape is expected to expand with highly targeted agents, such as monoclonal antibodies directed at discrete B-cell maturation stages or small molecules that interrupt B-cell activation pathways. Collectively, these precision strategies herald a rapidly evolving era for MuSK-MG, in which novel therapeutics grounded in pathophysiology continue to emerge and translate into tangible hope for patients.

## 5 Discussion

become a realistic objective.

MuSK-MG, an autoimmune disorder driven by anti-MuSK antibodies, has seen substantial advances in recent years in terms of pathogenic mechanisms, diagnostic detection, and targeted therapeutic strategies.

A relatively well-established consensus is that IgG4 anti-MuSK antibodies constitute the principal pathogenic basis of MuSK-MG. Through Fab-arm exchange, these antibodies become functionally monovalent and disrupt the interaction between LRP4 and MuSK, thereby impairing the Agrin–LRP4–MuSK signaling axis and ultimately leading to defective AChR clustering and endplate instability (17, 18). This mechanism largely explains the pathological and clinical distinctions between MuSK-MG and AChR-MG. However, the biology of MuSK antibodies is not limited to IgG4 alone. In addition to IgG4, IgG1–3 subclass antibodies may also be pathogenic, although their precise mechanisms of action remain controversial, including whether they involve receptor internalization, interference with noncanonical signaling pathways, disruption of AChR microcluster formation, or complement participation. Several factors may account for these discrepancies. First, the antibody sources used across studies differ substantially, including total patient IgG, purified IgG4 subclass antibodies, recombinant monoclonal antibodies, and engineered divalent antibodies, all of which may vary significantly in valency, affinity, and functional effects (51, 55, 58). Second, different antibodies target distinct MuSK domains. Antibodies directed against the Ig-like 1 domain are more commonly associated with the classical antagonistic effect, whereas antibodies recognizing other domains, particularly the Fz-CRD domain, may exert more complex or even apparently agonist-like effects (31, 48, 49). Third, the experimental models themselves may influence the conclusions. *In vitro* C2C12 myotube systems, passive transfer animal models, and NMJ models with different patterns of innervation differ in downstream signal amplification, receptor clustering dynamics, and endplate maturation. Accordingly, future studies should move beyond the binary question of whether anti-MuSK antibodies are pathogenic and instead establish a more refined correlation among antibody subclass, targeted epitope, functional effect, and clinical phenotype.

Anti-MuSK antibody testing is the cornerstone of MuSK-MG diagnosis; however, methodological differences markedly affect its sensitivity. RIPA remains the gold standard; however, its reliance on radioisotopes has limited its widespread use. ELISA offers convenience at the cost of lower sensitivity and a tangible risk of false-negatives, making it suitable for screening but not for definitive diagnosis. CBA and, more recently, L-CBA have shown substantially higher sensitivity, especially for refractory cases that return negative results on RIPA or ELISA, boosting detection rates by approximately 8%.

From a therapeutic perspective, the most important clinical implication of MuSK-MG is that optimal management should center on early control of the pathogenic B cell–antibody axis. Compared with AChR-MG, acetylcholinesterase inhibitors and thymectomy have limited roles in MuSK-MG, whereas B cell–targeted therapies such as rituximab show higher response rates and a clearer mechanistic rationale (97). FcRn inhibitors provide a new strategy for rapid intervention by reducing circulating IgG levels, while CD19-, CD38-, and BAFF-related approaches may further expand the scope of deeper B-cell-lineage-directed interventions (81, 98, 99). More advanced strategies, including MuSK agonist antibodies and CAAR-T cell therapy, represent two emerging directions: pathway restoration and etiologic elimination, respectively. Although most of these newer approaches remain in the early stages of development, they collectively reflect a shift in the treatment of MuSK-MG from empirical immunosuppression toward precision immunological intervention.

Despite the rapid progress in MuSK-MG research, several limitations remain in the field, including the relatively small size of patient cohorts, substantial heterogeneity in detection methodologies, the continued reliance of many functional studies on *in vitro* or animal models, and the fact that evidence for several emerging therapies is still largely preclinical. Future efforts should focus on establishing multicenter, prospective, standardized research systems that integrate multidimensional data, including antibody subclasses, targeted domains, functional activity, clinical phenotypes, and treatment outcomes, in order to build a mechanism-based stratification and prognostic framework for MuSK-MG. Only on this basis can the diagnosis of MuSK-MG move beyond the mere presence of antibodies toward functional antibody profiling, and treatment advance from symptom control to truly precise management.

Future research should prioritize several key directions. First, multicenter, prospective, standardized clinico-experimental cohorts should be established. Second, diagnostic platforms that combine sensitivity with mechanistic stratification should be developed, thereby shifting antibody testing from determining whether antibodies are present to defining what functional properties they possess. Third, the pathogenic roles of IgG1–3 subclass antibodies and antibodies targeting noncanonical epitopes should be further clarified. Fourth, models that more closely recapitulate the human disease state are needed to validate the optimal settings for emerging therapies, including MuSK agonist antibodies, combination strategies involving FcRn inhibitors, and CAAR-T cells. Only through such efforts can the diagnosis of MuSK-MG evolve from qualitative detection to mechanism-based stratification, and its treatment progress from empirical management to genuinely individualized precision intervention.
